# MicroRNA-210 suppresses glucocorticoid receptor expression in response to hypoxia in fetal rat cardiomyocytes

**DOI:** 10.18632/oncotarget.17801

**Published:** 2017-05-11

**Authors:** Shannalee R. Martinez, Qingyi Ma, Chiranjib Dasgupta, Xianmei Meng, Lubo Zhang

**Affiliations:** ^1^ Center for Perinatal Biology, Division of Pharmacology, Department of Basic Sciences, Loma Linda University School of Medicine, Loma Linda, CA 92350, USA

**Keywords:** hypoxia, microRNA-210, glucocorticoid receptor, cardiomyocyte, fetal programming

## Abstract

Hypoxia is a common intrauterine stressor, often resulting in intrauterine growth restriction and increased risk for cardiovascular disease later in life. The aim of this work was to test the hypothesis that microRNA-210 (miR-210) mediates the detrimental suppression of glucocorticoid receptor (GR) in response to hypoxia in fetal rat cardiomyocytes. Cardiomyocytes isolated from gestational day 21 Sprague Dawley fetal rats showed increased miR-210 levels and reduced GR abundance after exposure to *ex vivo* hypoxia (1% O2). In regard to mechanisms, the different contributions of hypoxia response elements (HREs) motifs in the regulation of miR-210 promoter activity and the miR-210-mediated repression of GR expression were determined in rat embryonic heart-derived myogenic cell line H9c2. Moreover, using a cell culture-based model of hypoxia-reoxygenation injury, we assessed the cytotoxic effects of GR suppression under hypoxic conditions. The results showed that hypoxia induced HIF-1α-dependent miR-210 production, as well as miR-210-mediated GR suppression, in cardiomyocytes. Furthermore, inhibition or knockdown of GR exacerbated cell death in response to hypoxia-reoxygenation injury. Altogether, the present study demonstrates that the HIF-1α-dependent miR-210-mediated suppression of GR in fetal rat cardiomyocytes increases cell death in response to hypoxia, providing novel evidence for a possible mechanistic link between fetal hypoxia and programming of ischemic-sensitive phenotype in the developing heart.

## INTRODUCTION

Cardiovascular disease is the leading cause of death in the United States and worldwide [1, 2]. Hypoxia, one of the most common insults during intrauterine life, is caused by conditions including pregnancy at high altitude, maternal anemia, pulmonary or heart disease, preeclampsia, drug abuse, or placental insufficiency [3]. Studies in humans and animals have linked hypoxia with intrauterine growth restriction (IUGR) and adult risk for cardiovascular disease [4–9], while the exact mechanisms underlying hypoxia-induced fetal programming of the heart are not yet known.

Growing evidence suggests that multi-factorial processes, including glucocorticoid receptor (*NR3C1*, GR) signaling pathways, involve in cardiomyocyte loss, reduced cardiomyocyte proliferation, increased cardiomyocyte hypertrophy, and altered gene expression patterns under hypoxia conditions [8, 10–14]. Under physiological conditions, the action of glucocorticoids through the GR is essential for proper cardiomyocyte development as well as overall cardiac function [15, 16]. Furthermore, glucocorticoids confer cardioprotection during myocardial injury [17–21]. Thus, alterations in GR expression during development may fundamentally reprogram cardiac structure and long-term function, including responsiveness to potentially beneficial therapeutic intervention. We have previously demonstrated a sustained reduction in GR expression in fetal, neonatal, and adult rat hearts in response to prenatal hypoxia through GR gene promoter hypermethylation and epigenetic repression in the developing heart [22, 23]. And the long-term nature of GR suppression following hypoxic insult *in utero* suggests epigenetic mechanisms in the regulation of GR expression. However, the epigenetic regulation of GR expression at transcription/translation level remain largely elusive.

MicroRNAs (MiRs) are a type of non-coding RNAs with about 21–22 nucleotides in length whose mature form binds to a discrete region in the 3′-untranslated region (3′-UTR) of target gene mRNA, leading to the suppression of target gene expression. A growing list of hypoxia-responsive miRs have been identified [24]. Among these, the most consistent and powerful response to hypoxia is observed in miR-210 activity. MiR-210 is directly linked to hypoxia through its promoter that is driven by nuclear accumulation of hypoxia-inducible factor (HIF)-1α. Because of its robust response to hypoxia and its role in mediating multiple physiological processes, miR-210 has been termed the “master hypoxamir” [25]. The activity of miR-210 has been linked to the mitochondrial metabolism, angiogenesis, cell proliferation, cell differentiation, apoptotic cell death, *etc* [25–27]. However, its effects on the fetal programming of the cardiovasculature are only beginning to be understood. Our recent study provided the first evidence that miR-210 repressed GR expression in the neonatal brain of rats after hypoxic-ischemic brain injury [28]. These findings, together with the substantial body of evidence regarding the cardioprotective effects of glucocorticoids in cardiac ischemic injury, suggesting a potential role of miR-210 in hypoxia-induced fetal programming of the heart by modulating GR expression.

In the present study, we reveal the role of HIF-1α-dependent miR-210-mediated suppression of GR in cardiomyocyte cell death in response to hypoxia. We report that hypoxia drives miR-210 production in a HIF-1α-dependent manner, and that this event is directed by a single hypoxia response element (HRE) within the miR-210 promoter. Furthermore, we identify the GR as a downstream target of miR-210, demonstrating a negative regulation of miR-210 on the expression of the GR by binding to the 3′-UTR of its transcript. Finally, we determine that the suppression of the GR under hypoxic conditions results in cardiomyocyte demise by apoptosis. Taken together, these findings uncovers a new mechanism of GR regulation by miR-210 in hypoxia-induced programming of ischemic-sensitive phenotype in the developing heart.

## RESULTS

### Hypoxia induces miR-210 expression and suppresses GR abundance in fetal rat hearts

**Figure 1:**
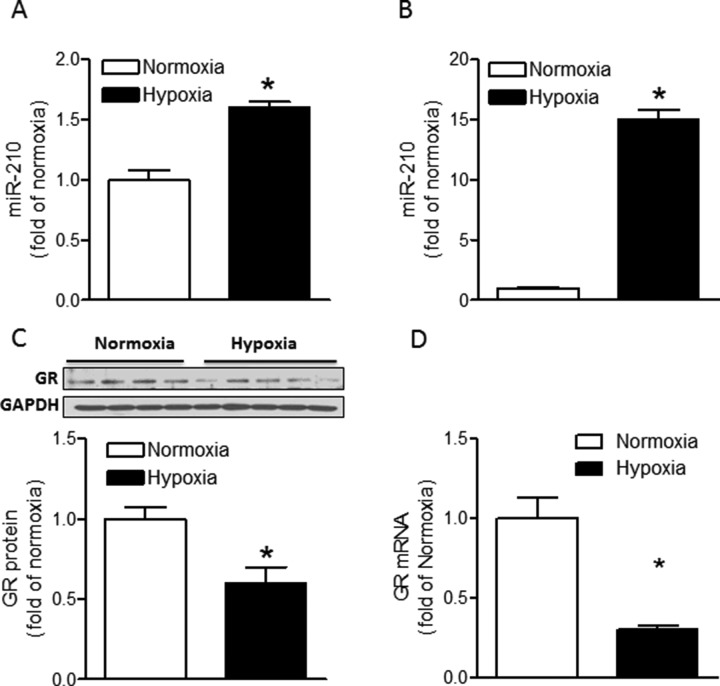
Hypoxia induces miR-210 expression and suppresses GR expression in fetal rat hearts (**A**) Hearts were isolated from E21 fetuses from pregnant rats treated with normoxia or hypoxia at 10.5% O_2_ from day 15 to day 21 of gestation, and miR-210 expression was measured by miScript miR real-time RT-qPCR. *n* = 8. (**B**, **C**, **D**) Cardiomyocytes isolated from E21 fetuses were treated with normoxia or hypoxia (1% O_2_) for 24 hours. MiR-210 expression was measured by miScript miR real-time qRT-PCR (B), *n* = 6; GR protein abundance was measured by Western blot (C), *n* = 4–5, and GR mRNA abundance was determined by real-time qRT-PCR (D), *n* = 6. Data are mean ± SEM. *p < 0.05, hypoxia *vs*. normoxia.

Maternal hypoxia caused a 59% increase (*p* < 0.05) in miR-210 expression in fetal rat hearts compared to fetal hearts from normoxia control animals (Figure 1A). To determine the direct effect of hypoxia, cardiomyocytes were isolated from fetal rat hearts and were treated *ex vivo* with hypoxia (1% O_2_) for 24 hours. As shown in Figure 1B, hypoxia stimulated a 14-fold increase in miR-210 expression in fetal cardiomyocytes. Moreover, we found that the direct effect of hypoxia treatment of fetal cardiomyocytes resulted in a significant decrease in GR protein (Figure 1C) and mRNA abundance (Figure 1D).

### Characterization of the rat miR-210 promoter

**Figure 2:**
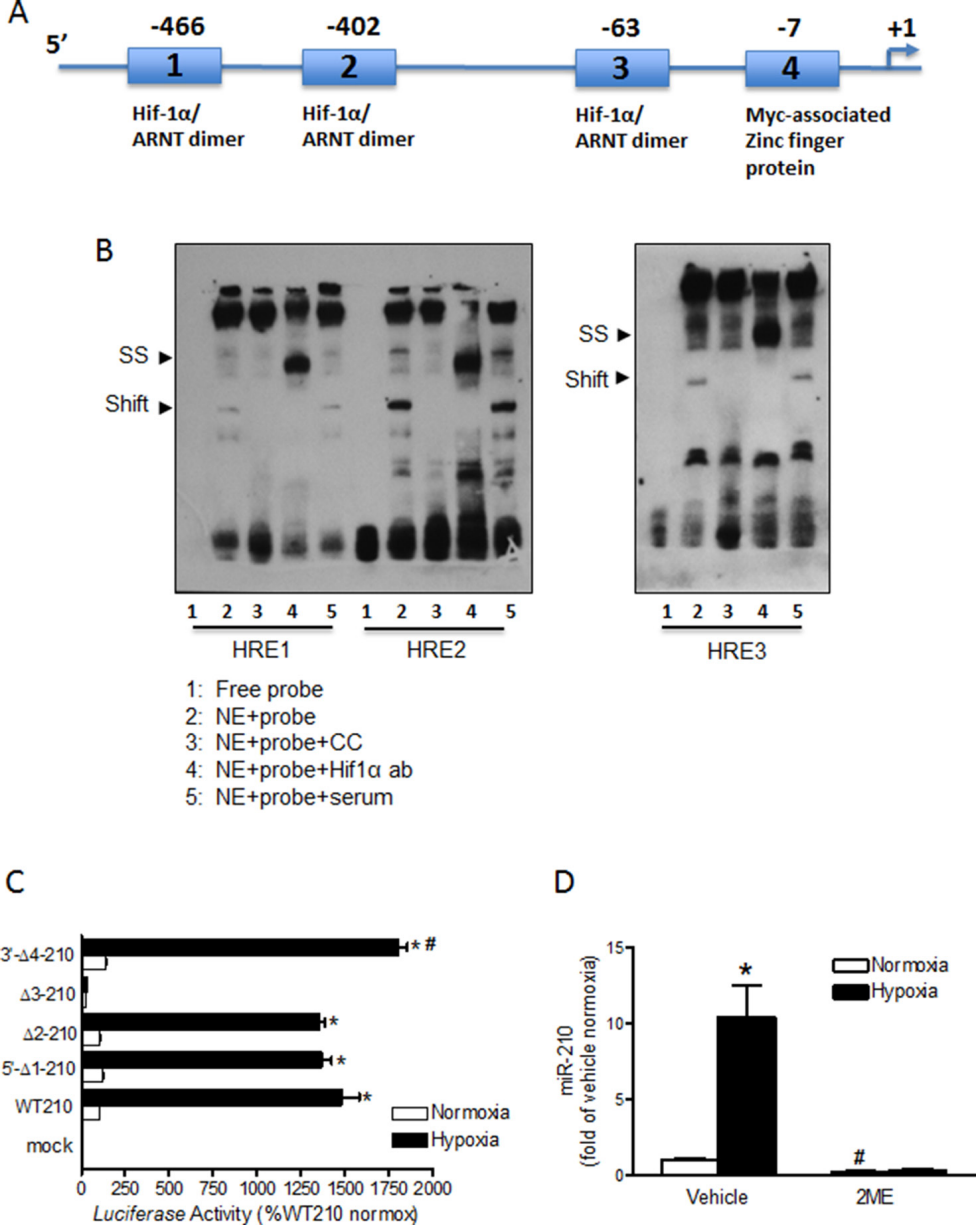
Characterization of the miR-210 promoter (**A**) Schematic representation of the miR-210 promoter, indicating putative binding sites of HIF-1α/ARNT dimer in three HRE motifs (1, 2 and 3). The fourth motif is where Myc-associated zinc finger related transcription factor binds. Nucleotide numbers indicate position of core elements in these sites. (**B**) EMSA was performed with nuclear extracts (NE) from fetal hearts and biotin labeled ds-oligo probes containing HRE 1, 2 and 3, respectively: HRE1-S: 5′-aggcagaggcgtacgtgcctcccgaggg; HRE1-AS: 5′-ccctcgggaggcacgtacgcctctgcct; HRE2-S: 5′-attgccaggaatacgtgcgcctggagag; HRE2-AS: 5′-ctctccaggcgcacgtattcctggcaat; HRE3-S: 5′-ctccccaagccgacgtgcagaaaagaac; HRE3-AS: 5′- gttcttttctgcacgtcggcttggggag. Unlabeled ds-oligos were used for cold competition (CC) experiments. For super-shift (SS) experiments, NE were incubated with biotin labeled ds-oligo probes in the presence of either anti-HIF-1α or normal rabbit serum. (**C**) Reporter gene constructs of wild-type miR-210 (WT210) promoter-pGL3 and four site-specific deletion constructs of HRE 1–4 (Δ1 through Δ4) were transiently co-transfected along with pRLSV40-*Luc* in H9c2 cells in normoxic and hypoxic (1% O_2_) environments. *Firefly* and *R. reniformis* luciferase activities in cell extracts were measured using a dual-luciferase reporter assay system. The promoter activities were calculated by normalizing the *firefly* luciferase to *R. reniformis* luciferase activity. Data are mean ± SEM. *p < 0.05, hypoxia *vs*. normoxia; ^#^*p <* 0.05, Δ4–210 *vs*. WT210 in hypoxia; *n* = 6. (**D**) Cardiomyocytes were isolated from E21 fetal rats and treated with normoxia or hypoxia (1% O_2_) in the presence of 2ME or vehicle for 24 hours. MiR-210 expression was determined by miScript miR real-time RT-PCR. Data are mean ± SEM. *p < 0.05, hypoxia *vs*. normoxia; ^#^*p <* 0.05, 2ME *vs*. vehicle in normoxia; *n* = 7.

In the rat, the miR-210 coding gene is located on chromosome 1. Using the Genbank and miRbase as our points of reference we were able to locate the promoter region of miR-210 on rat chromosome 1. A bioinformatics (Genomatix) transcription factor search of the 998-bp segment upstream of rat pri-miR-210 transcription start site revealed three potential hypoxia response elements (HREs) at −466, −402, −63 (Supplementary Figure 1; Figure 2A). The binding of HIF-1α to these HREs were evaluated by EMSA and super-shift assays. As shown in Figure 2B, incubation of fetal heart nuclear extracts (NE) with biotinylated double-stranded oligonucleotide probes representing HREs 1, 2, and 3 resulted in a shift of DNA-protein complexes, which were further super-shifted by an anti-HIF-1α antibody.

### Function of HREs in miR-210 promoter activity

The relative contribution of each of the three HRE motifs in the regulation of miR-210 promoter activity was tested by site specific deletions (Δ1 through D4) in reporter gene assays along with the WT-miR-210 pGL3; in normoxic and hypoxic (1% O_2_) environments for 26 hours in parallel. As shown in Figure 2C, compared to normoxic control there was a robust stimulation of WT-pGL3-miR-210 promoter activity by 15 folds in *ex vivo* hypoxic treatment. Deletions of HRE1 and HRE2 resulted in no significant loss of miR-210 promoter activity in hypoxia. Thus, despite deletions of HRE1 and HRE2 motifs, the miR-210 promoter activity in hypoxia remained upregulated to at least 13 fold. In sharp contrast, deletion of only HRE3 caused complete loss of promoter activity in hypoxia. As a result, there was no significant difference between miR-210 promoter activities under normoxic versus hypoxic conditions when HRE3 was deleted. Interestingly, compared to WT-pGL3-miR-210 promoter activity in hypoxia, deletion of site 4 resulted in a slight (20%) but significant increase of promoter activity in hypoxia. This finding possibly indicates a de-repression of miR-210 promoter activity that is induced when site 4 is deleted.

### Hypoxia-induced miR-210 expression depends on HIF-1α activity

To assess the role of HIF-1α in hypoxia-induced miR-210 expression in cardiomyocytes, we administered 2ME, an inhibitor of HIF-1α nuclear accumulation [29, 30], during *ex vivo* hypoxic treatment in primary fetal cardiomyocytes. Inhibition of HIF-1α nuclear accumulation abolished miR-210 production in response to hypoxia (Figure 2D). Of interest, 2ME treatment also reduced miR-210 production under normoxic conditions by 77% compared to vehicle-treated normoxic cells (Figure 2D). These data indicate that HIF-1α is an essential determinant of miR-210 expression under normoxic conditions as well as in response to hypoxia.

### miR-210 directly binds to the 3′-UTR of GR mRNA

**Figure 3:**
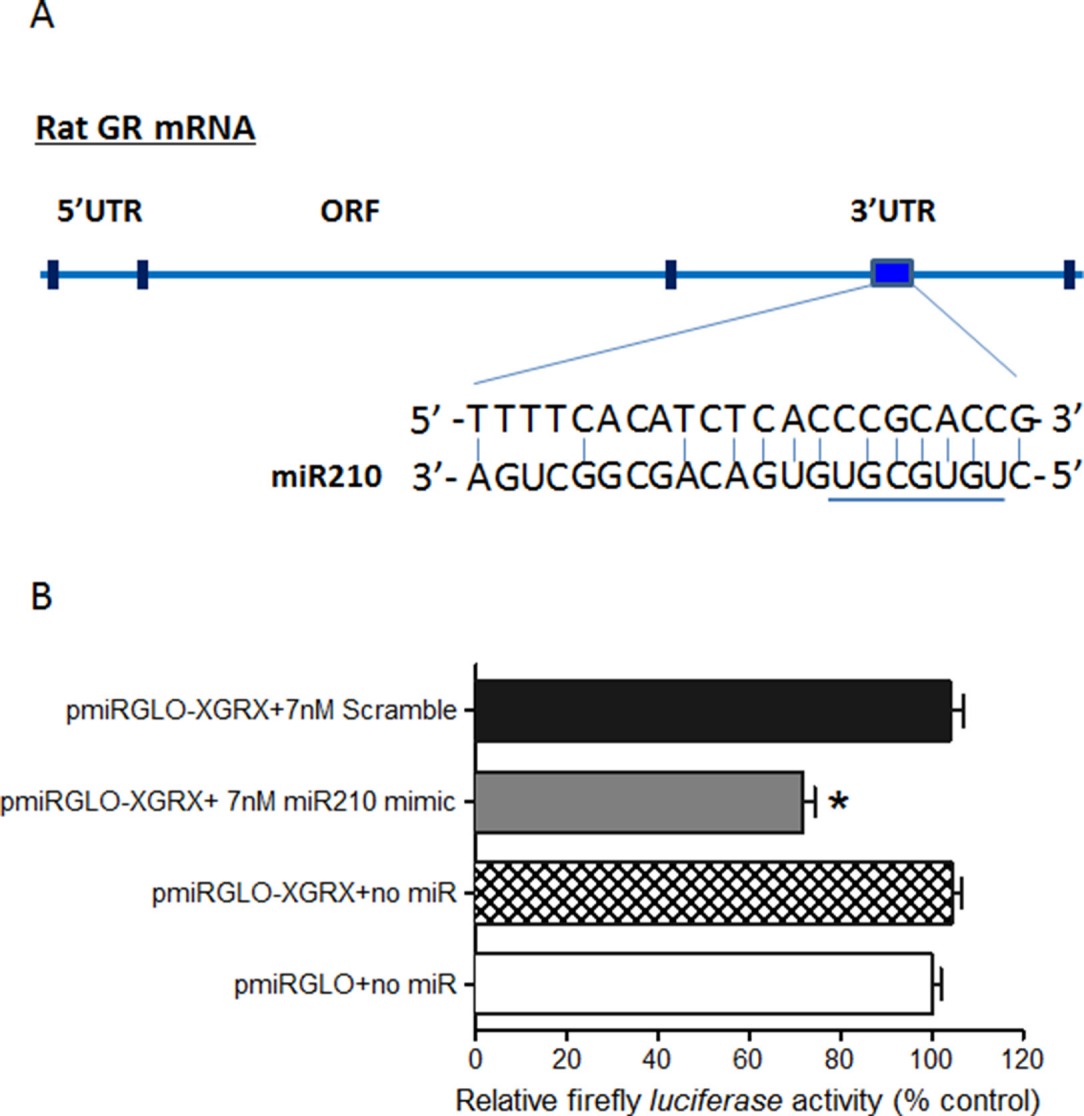
MiR-210 directly binds to the 3′-UTR of GR mRNA (**A**) Schematic illustrating sequence complementarity of the seed sequence of rat miR-210 to the 3′-UTR of GR mRNA. (**B**) H9c2 cells were transfected with pmiRGLO vector containing putative miR-210 binding sites within the GR 3′-UTR (XGRX) and co-transfected with either miR-210 mimic or negative control scramble for 48 hours. Luciferase activity was measured. Data are mean ± SEM. *p < 0.05, pmiRGLO-XGRX + 7 nM miR210 mimic *vs*. pmiRGLO-XGRX + 7 nM scramble; *n* = 6.

The mature miR-210 has significant sequence complementarity of its seed region to a single discrete nucleotide sequence in the 3′-UTR of GR mRNA (Figure 3A). Thus, luciferase assays were used to determine the effect of miR-210 in downregulating GR expression in the rat cardiomyocyte cell line H9c2. Cells transfected with control vector only (pmiRGLO) or that contained target region of GR mRNA 3′-UTR (pmiRGLO-XGRX) expressed baseline (100%) levels of luciferase activity. In contrast, cells that were co-transfected with pmiRGLO-XGRX plus miR-210 mimic (pmiRGLO-XGRX + 7 nM miR-210 mimic) had 30% inhibition of luciferase activity compared to cells co-expressing the GR 3′-UTR and a scrambled mimic (pmiRGLO-XGRX + 7nM scramble) (Figure 3B). Thus, miR-210 suppressed the Firefly luciferase gene expression by binding to the miR-210 target region of GR mRNA 3′-UTR, indicating that the GR transcript is a valid target of miR-210.

### miR-210 suppresses GR expression in cardiomyocytes under normoxic or hypoxic conditions

**Figure 4:**
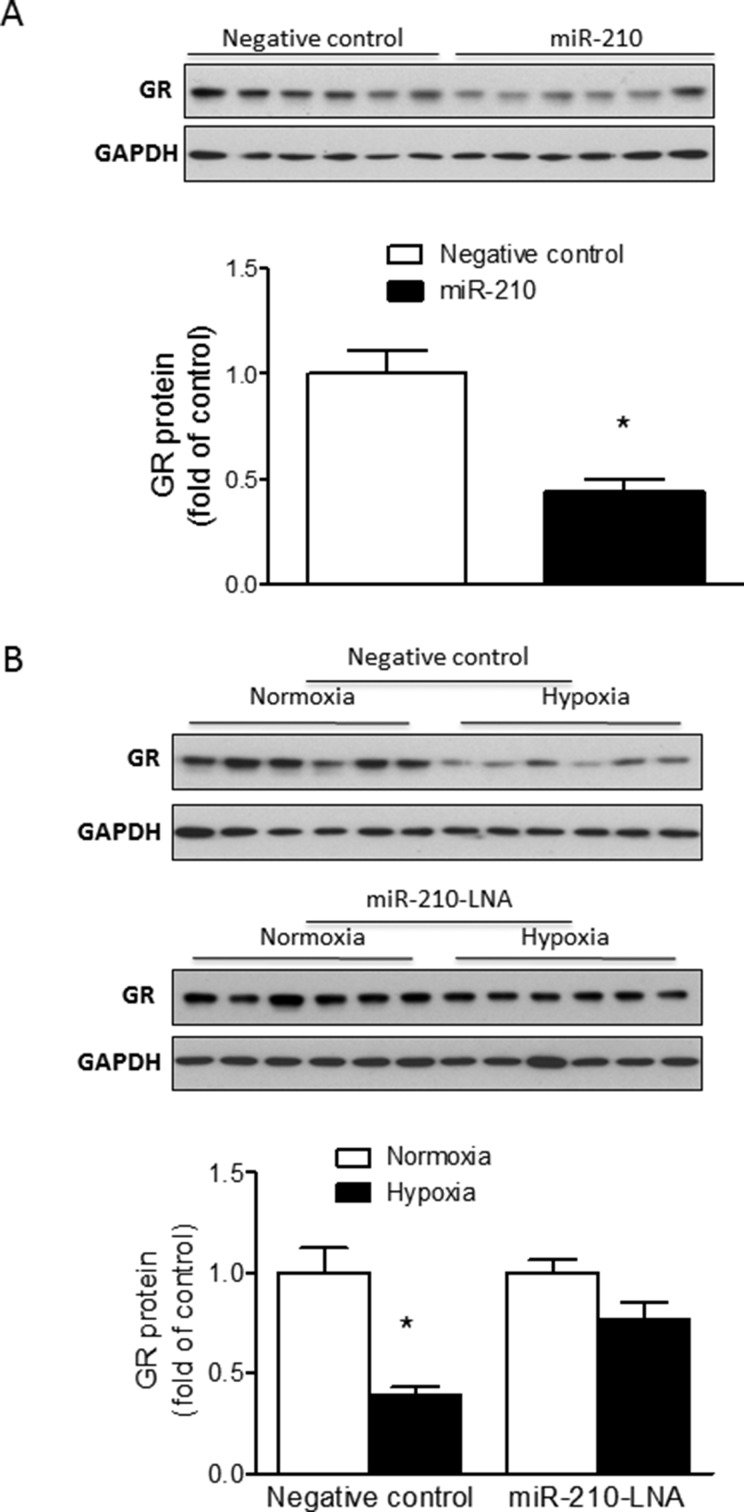
MiR-210 reduces GR protein abundance in fetal rat cardiomyocytes Cardiomyocytes were isolated from E21 fetal rat hearts. (**A**) Cells were treated with 100 nM miR-210 mimic or negative control for 48 hours; (**B**) Cells were treated with normoxia or hypoxia (1% O_2_) in the presence of 50 nM miR-210-LNA or negative control for 24 hours. GR protein abundance was measured by Western blot. Data are mean ± SEM. *p < 0.05, miR-210 mimic *vs*. negative control in (A), and hypoxia *vs*. normoxia in (B); *n* = 6.

To further determine the inhibitory effect of miR-210 on endogenous GR expression in cardiomyocytes, primary fetal cardiomyocytes were treated with miR-210 mimic or negative control (100 nM) under normoxic conditions for 48 hours followed by detection of GR protein abundance by Western blot. Consistent with the finding of luciferase assays described above, miR-210 treatment significantly reduced GR protein abundance in fetal cardiomyocytes compared to cells treated with negative control (Figure 4A).

To determine the influence of miR-210 on GR expression under hypoxic conditions, cardiomyocytes were treated with miR-210-LNA or negative control (50 nM) under normoxic or hypoxic (1% O_2_) conditions for 24 hours followed by detection of GR protein expression by Western blot. As compared to the negative control in which hypoxia resulted in a significant decrease of GR protein abundance, miR-210-LNA blocked the hypoxia-induced reduction of GR protein in cardiomyocytes (Figure 4B).

### HIF-1α inhibition abrogates hypoxia-induced suppression of GR

**Figure 5:**
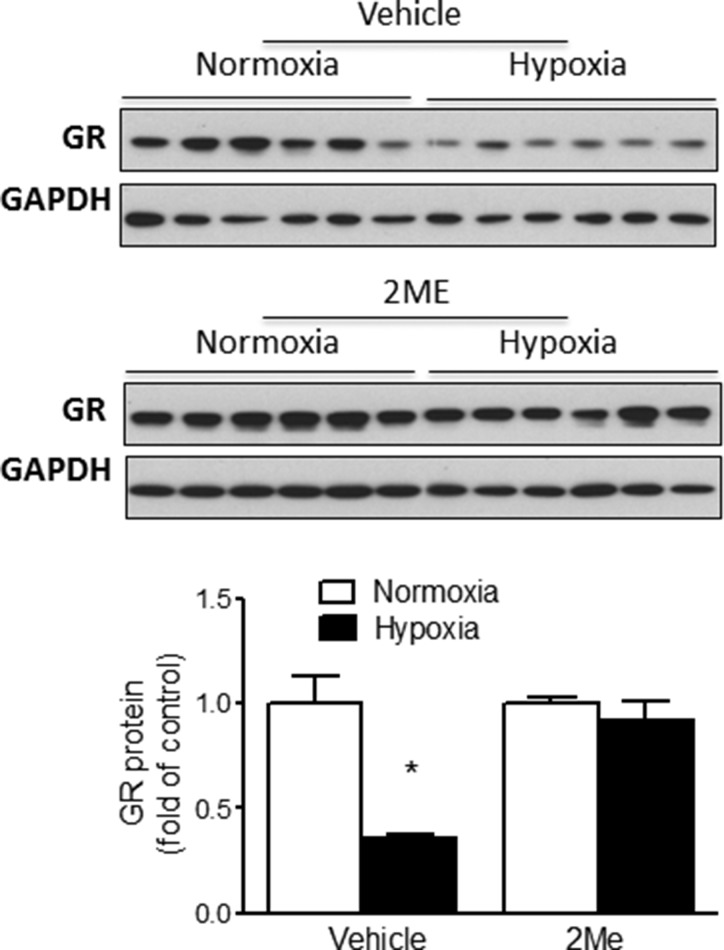
HIF-1α regulates hypoxia-mediated suppression of GR in fetal rat cardiomyocytes Cardiomyocytes were isolated from E21 fetal rat hearts and were treated with normoxia or hypoxia (1% O_2_) in the presence of 2ME or vehicle for 24 hours. GR protein abundance was measured by Western blot. Data are mean ± SEM. * *p <* 0.05, hypoxia *vs*. normoxia; *n* = 6.

Given that HIF-1α played a critical role in the regulation of hypoxia-induced miR-210 expression (Figure 2D), we further determined the role of HIF-1α in hypoxia-mediated suppression of GR protein expression. Fetal cardiomyocytes were treated with normoxic (21% O_2_) or hypoxic (1% O_2_) conditions for 24 hours in the absence or presence of 2ME followed by detection of GR expression by Western blot. As shown in Figure 5, in the absence of 2ME, hypoxia significantly reduced GR expression in cardiomyocytes. In contrast, inhibition of HIF-1α nuclear accumulation by 2ME abolished the hypoxia-induced reduction in GR protein expression. This finding is consistent with the notion that HIF-1α mediates GR suppression by hypoxia in fetal cardiomyocytes.

### Inhibition of the GR exacerbates cell death caused by hypoxia

**Figure 6:**
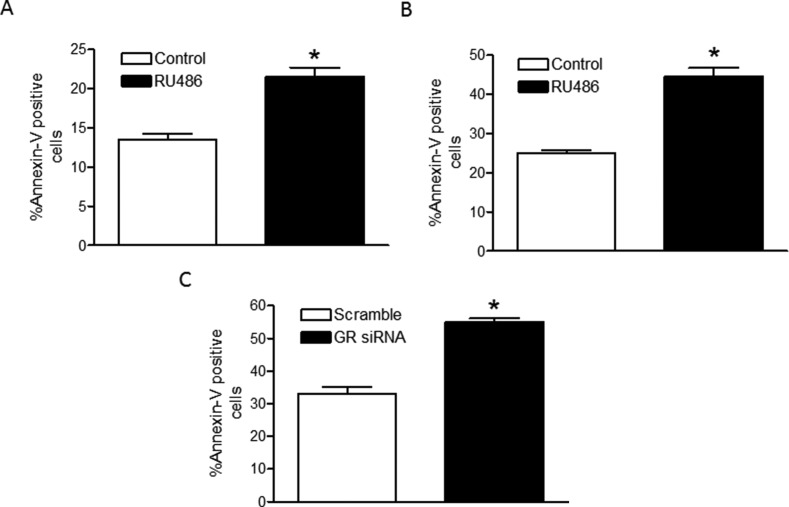
Inhibition of the GR increases cardiomyocyte death by hypoxia Primary fetal cardiomyocytes were treated with 1 μM RU486 (**A**), *n* = 6; and H9c2 cells were treated with 1 μM RU486 (**B**); *n* = 5–6, or transfected with two GR siRNA oligos (**C**), *n* = 8; prior to the hypoxia-reoxygenation treatment. Cardiomyocyte apoptosis was determined with Annexin-V staining using the Annexin-V-FITC Apoptosis kit for flow cytometry. Data are mean ± SEM. *p < 0.05, hypoxia *vs*. normoxia.

To determine the effects of hypoxia-induced GR suppression on the viability cardiomyocytes, we used pharmacological (RU486; mifepristone) and molecular (siRNA-mediated knockdown) inhibition of GR under hypoxic conditions followed by assessment of myocyte apoptosis using Annexin-V staining and analysis by flow cytometry. In primary fetal cardiomyocytes, GR inhibition by RU486 significantly increased hypoxia-induced apoptosis (Figure 6A). Similarly in H9c2 cells, RU486 significantly increased hypoxia-induced apoptosis (Figure 6B). Next, we validated the specificity of GR-mediated effect by knockdown of the GR using siRNAs designed to target rat GR. As shown in Supplementary Figure 2A, 2B, two separate GR siRNA oligos significantly decreased GR protein and mRNA abundance in H9c2 cells. Of importance, knockdown of the GR significantly increased hypoxia-induced apoptosis (Figure 6C), demonstrating a critical role of GR in protecting cardiomyocytes from hypoxia-reoxygenation injury.

## DISCUSSION

The present study presents several novel findings. First, we characterize the miR-210 promoter for the first time in the fetal heart of rat and identify the HRE_-63_ as the HIF-1α binding site responsible for the robust induction of the miR-210 promoter in response to hypoxia. Second, we also provide the novel evidence that GR mRNA is a direct downstream target of miR-210 and that hypoxia suppresses the GR in a HIF-1α- and miR-210-dependent manner in the fetal rat heart. Of importance, we demonstrate a critical role of endogenous GR in protecting cardiomyocytes from hypoxia-reoxygenation injury. These findings provide important original insights into epigenetic mechanisms in the regulation of GR expression patterns in the developing heart, contributing to our understanding of fetal stress-mediated developmental programming of cardiovascular disease.

Consistent with previous studies in neonatal rat cardiomyocytes [31], we found that hypoxia induced miR-210 expression in fetal rat cardiomyocytes. Furthermore, we presented novel evidence that maternal hypoxia during gestation induced miR-210 expression in fetal rat hearts, implicating miR-210 as a possible mediator of fetal stress-mediated programming in the heart. We also demonstrated that *ex vivo* hypoxia in cultured primary fetal cardiomyocytes resulted in reduction in GR protein and mRNA levels. This is consistent with our prior findings that hypoxia *in utero* led to reduction in both GR protein and mRNA levels in the fetal heart [22, 23, 32]. Mechanistically, we have demonstrated that the hypoxia-induced hypermethylation of GR promoter resulted in the suppression of GR expression in the developing heart following hypoxia [23]. However, whether other epigenetic mechanisms are also involved in the GR regulation after hypoxia is still unclear. Bioinformatics analysis for miR-210 targets in databases (miRWalk 2.0) and our recent study [28] showed that GR was a potential downstream target of miR-210, implicating that, in addition to promoter hypermethylation, hypoxic-induced upregulation of miR-210 may also contribute to the downregulation of GR expression in cardiomyocytes in response to hypoxia.

We then determine the promoter-level regulation of miR-210 in rat cardiomyocytes in response to hypoxia. By bioinformatics search, we identified putative HRE sites for HIF-1α/ARNT dimer binding *via* (Supplementary Figure 1). The core DNA sequence necessary for HIF-1α/ARNT binding is 5′-(A/G) CGTG-3′ [33, 34] and is termed the HIF-1 binding site (HBS). In many hypoxia-inducible genes, the HRE also contains a HIF-1 ancillary sequence (HAS) in addition to the HBS [35]. HAS sequences may be located 8–9 nucleotides upstream or downstream of the HBS and work with the HBS to facilitate transcription activation [34]. In the present study, we demonstrated that the three putative HREs of rat miR-210 are of the HBS type and HIF-1α is capable of binding to all three HRE sites identified within the miR-210 promoter. These sites were then evaluated together with a fourth site, a non-HIF-1α/ARNT binding site, in reporter gene assays using site-specific deletion constructs. Hypoxia induced a robust increase in miR-210 promoter activity in cells containing the intact wild type miR-210 promoter. The deletion of HRE_-63_ (HRE3), but not others, completely silenced the reporter construct in hypoxia. These findings suggest that of the three HREs, HRE_-63_ is the sole determinant of miR-210 promoter activity in response to hypoxic insult in cardiomyocytes. Our findings are in agreement with the previous findings in mouse and human showing that miR-210 promoter harbors three HREs and the HRE3 is identified as the HIF-1α binding site responsible for the robust induction of miR210 promoter activity [36, 37]. Moreover, we found that deletion of site 4, which is not a HIF-1α but a myc-associated zinc finger protein binding site, resulted in the upregulation of hypoxia-induced miR-210 level, as compared to cells with intact miR-210 promoter. This finding suggests that site 4 may be an important repressor of miR-210 expression, which restricts miR-210 overexpression in response to hypoxia. The promoter assay also confirmed that miR-210 is a HIF-1α-dependent gene but is not HIF-2α responsive [37]. These studies suggest that the miR-210 promoter is highly conserved across species among humans and rodents. In addition, we observed a significant reduction in promoter activity under normoxic conditions in cells lacking HRE3 compared to cells with the wild type miR-210 promoter. This demonstrates that HRE3 also drives baseline endogenous expression of miR-210 in cardiomyocytes. This finding was validated in primary fetal cardiomyocytes by using 2ME, which is a well-known HIF-1α inhibitor by blockage of HIF-1α protein synthesis [38] and HIF-1α transcriptional activities [39]. We found that HIF-1α inhibition blocked miR-210 production in response to hypoxia in cardiomyocytes. Interestingly, 2ME also suppressed miR-210 production under normoxic conditions by 77% compared to vehicle-treated cells, which is consistent with the finding of the reporter gene assays of miR-210 promoter activity. Taken together, we conclude that HIF-1α binding to HRE3 in the miR-210 promoter is the major determinant of miR-210 production in cardiomyocytes, both under normoxic and hypoxic conditions. These findings are consistent with previous reports that HIF-1α drives miR-210 production under hypoxic conditions as well as baseline miR-210 expression under normoxic conditions in cancer cells [37, 40, 41] and in differentiating myoblasts [36].

To validate that miR-210 negatively regulates GR expression, we conducted *in vitro* UTR analyses using luciferase reporter gene assays. The result showed that miR-210 mimic significantly reduced reporter activity in the cells containing the target sequences of GR 3′UTR. Similar approaches have been applied to identify other miRs that target the GR transcript, including miR-124, -142, -101a, -433, and -96 [42–45]. The mature miR-210 has significant sequence complementarity of its seed region at the 5′ end (nucleotides 2–8) to the 3′UTR of GR mRNA with an exact match of positions 3–7 in the mature miR-210 to GR 3′UTR. Thus, it is highly likely that the mutation of complementary nucleotides at GR 3′UTR will abrogate binding and function of miR-210 with regards to GR mRNA. Moreover, the functional significance of negative regulation of GR expression by miR-210 was demonstrated in primary fetal cardiomyocytes showing that miR-210 mimic treatment significantly reduced GR protein abundance. And miR-210 inhibition by its locked nucleic acid (LNA) inhibitor reversed hypoxia-induced suppression of GR protein in primary fetal cardiomyocytes. The LNA is chemically modified *via* a methylene linker that connects the 2′ *O*-oxygen to the 4′ position and are designed for optimal cellular uptake, stability *in vivo*, and highly specific binding to target miRs [46]. Our previous study has determined that miR-210-LNA reduces brain miR-210 levels in a perinatal HI rat model [28]. Thus, all this evidence indicates that miR-210 is a negative regulator of GR in cardiomyocytes through interaction with the 3′-UTR of GR transcript. To confirm the role of HIF-1α in the miR-210-mediated suppression of the GR during hypoxia, we inhibited HIF-1α nuclear accumulation using 2ME and measured GR protein in cardiomyocytes. Compared to a significant reduction in GR protein expression in vehicle-treated cells in response to hypoxia, no significant change was detected in GR protein expression in 2ME-treated hypoxic cells. Taken together, our data indicate that HIF-1α works through miR-210 activity regulating GR expression under hypoxic conditions.

Our previous *in vivo* study demonstrated that maternal chronic hypoxic exposure during gestation decreased GR expression [23] and increased apoptosis in fetal rat heart [11]. Given the potential cardioprotective effects of glucocorticoid administration during hypoxic/ischemic injury *in vivo*, we set out to assess the functional relevance of GR suppression in hypoxia. Using a cell culture model of hypoxia-reoxygenation injury, we demonstrated that inhibition of the GR by RU486 caused increased apoptotic cell death in response to hypoxia-reoxygenation injury as detected by Annexin-V staining and flow cytometric analysis. This was true for both primary fetal cardiomyocytes and H9c2 cells. To further determine the specificity of GR-mediated effect, siRNA-mediated knockdown of the GR was performed to evaluate the effects of selective GR suppression on cardiomyocyte viability during hypoxia-reoxygenation injury. Consistent with pharmacological inhibition of the GR, molecular silencing of the GR resulted in a comparable increase in cell death during hypoxic treatment. These findings demonstrate a critical role of endogenous GR in protecting cardiomyocytes from hypoxia-reoxygenation injury. While mechanisms of cell death during hypoxic insult in cardiomyocytes have been well studied, the role of the GR during this process is less well understood. It is widely accepted that glucocorticoid exerts a biphasic response on most cell types, with low (physiologic) level signaling enhancing cell survival and high doses causing cytotoxicity [47]. It has been shown in both rat embryonic H9c2 cardiomyocytes and primary cardiomyocytes that serum deprivation triggered cardiomyocyte apoptosis is inhibited by dexamethasone [48]. Further studies in mice with cardiomyocyte-specific deletion of the GR revealed aberrant regulation of a large cohort of genes associated with cardiovascular disease as well as unique disease genes associated with inflammatory processes, and demonstrated that a deficiency in cardiomyocyte glucocorticoid signaling leads to spontaneous cardiac hypertrophy, heart failure, and death, indicating an obligate role for the GR in maintaining normal cardiovascular function [49].

Rodents have been widely used to investigate functionalities and mechanisms of GR signals in the development of cardiomyocytes in response to maternal stress, providing valuable information that are of clinical relevance. However, we cannot rule out the potential species difference of GR signals between humans and rodents. GR are activated by glucocorticoids (cortisol in humans and corticosterone in rodents), which are cholesterol-derived hormones secreted by the adrenal gland. The genomic structure and transcripts of GR gene between humans and rodents show high similarities [50, 51]. However, the proteins of GR gene encoded are slightly different due to the differential transcript splicing. In general, three isoforms of GR protein, GRα, GRβ and GRp, are expressed in humans while only one type of GR protein is thought to be expressed in rodents, which is corresponding to GRα in humans [51, 52]. Moreover, mechanisms of GR activation by glucocorticoids also show some degree of species-differential manner. For example, the sensitivity of GR after binding with glucocorticoids to kinase-mediated phosphorylation, such as glycogen synthase kinase beta (GSK3β), is different between humans and rodents, suggesting that GR in rodents may not fully take the receptor action of humans [53]. Thus, recognition of similarity and discrepancy of GR signals between humans and rodents would be benefit for translating these findings to humans.

In summary, the present study provides insights into the epigenetic mechanisms through which fetal hypoxia alters GR expression in cardiomyocytes by miR-210. We demonstrate that hypoxia reduces GR expression through a miR-210-dependent pathway, revealing a pivotal role for miR-mediated regulation in epigenetic programming of gene expression patterns in the heart. Together with our prior studies showing regulation of GR by alterations of DNA methylation levels in response by hypoxia, our finding advances the understanding of the epigenetic regulation of GR in the programming of ischemic-sensitive phenotype in the developing heart.

## MATERIALS AND METHODS

### Animal experiments

Time-dated pregnant Sprague-Dawley rats were purchased from Charles River Laboratories (Portage, MI). For maternal hypoxia studies, pregnant rats were exposed to room air (normoxic control) or hypoxia (10.5% O_2_) from gestational day 15 to day 21, as we described previously [54]. Experiments from our previous study have indicated that the ambient oxygen level of 10.5% lowers arterial Po_2_ of pregnant rats to approximately ~50 mmHg [55], which caused fetal hypoxia and upregulated hypoxia marker HIF-1α levels in fetal hearts [11]. For *ex vivo* hypoxia treatment in isolated cardiomyocytes, hearts were isolated from gestational day 21 fetuses and enzymatically digested using 0.1% trypsin and 0.5 mg/mL type II collagenase, as we described previously [56]. To isolate hearts, rats were anaesthetized with isoflurane (5% for induction and 3% for maintenance). The adequacy of anesthesia was determined by the loss of a pedal withdrawal reflex and any other reaction from the animal in response to pinching the toe, tail, or ear of the animal. After removing fetuses, pregnant rats were euthanized by cardiac exsanguination. All procedures and protocols in this study were in adherence with the *National Institutes of Health Guide for the Care and Use of Laboratory Animals* and were approved by the Institutional Review Board of Loma Linda University.

### Cardiomyocyte culture, transfection, and treatment

Cardiomyocytes isolated from gestational day 21 fetuses were seeded at 6.0–7.5 × 10^5^ cells per well of a 6-well plate and cultured in M199 medium (Hyclone, Logan, UT) supplemented with 10% fetal bovine serum (FBS; Gemini Bio-Products) and 1% penicillin/streptomycin at 37°C in 5% CO_2_ as previously described [56]. After a 24-hour recovery, Cells were then treated with 100 μM bromodeoxyuridine (BrdU, Sigma-Aldrich, St. Louis, MO) for 24 hours. At this time, cardiomyocytes were approximately 70–80% confluent in monolayer and spontaneously beating. Transfection of either miR-210 mimic or inhibitor was carried out using the HiPerFect transfection reagent (Qiagen Inc., Valencia, CA) according to the manufacturer's instructions. For transfection with miR-210 mimic, cells were cultured in antibiotic-free M199 supplemented with 10% FBS for 1 hour, and then transfected with 100 nM miR-210 mimic or its negative control (Qiagen) under normoxic conditions for 48 hours. For transfection with miR-210 inhibitor, cells were cultured in antibiotic-free M199 supplemented with 10% FBS for 1 hour, and then transfected with 50 nM miR-210 locked nucleic acid (miR-210-LNA; Exiqon) inhibitor or its negative control (Exiqon, Woburn, MA) under normoxic (21% O_2_) or hypoxic (1% O_2_) conditions for 24 hours. For treatment with 2-methoxyestradiol (2ME; Sigma-Aldrich, St. Louis, MO), an inhibitor of HIF-1α accumulation [29, 30], cells were cultured in fully supplemented M199 containing 3 μM 2ME under normoxic (21% O_2_) or hypoxic (1%) conditions for 24 hours. The 1% O_2_ (~7 mmHg) is commonly used to induce hypoxic treatment on tissue or cells [8, 54, 57, 58]. Our previous studies showed that 1% O_2_ induced cellular and molecular responses in cardiomyocytes such as the nuclear accumulation of HIF-1α [8, 54].

### Cloning of rat miR-210 promoter

The sequence information of the rat miR-210 promoter was obtained from Genbank (http://www.ncbi.nlm.nih.gov/gene/?term=100314053). In addition, miRbase describes the sequence information of the rat (*rno*) pre-miR-210 stem-loop and mature miR-210 (http://www.mirbase.org/cgi-bin/mirna_entry.pl?acc=MI00000950). A 1057 base pair (bp) rat miR-210 promoter sequence was amplified by preparative PCR from 100 ng rat genomic DNA using the following forward and reverse primers: forward: 5′-gagacccGCTAGCccagaagaggacatcagatcc, reverse: 5′-gagacccAAGCTTctgcagccagtgaacacg. After gel purification, the 1057 bp amplicon was cloned in pCR 4-TOPO vectors (Invitrogen, Carlsbad, CA) and confirmed by sequencing. For the purpose of the reporter gene assay, the ^5′^NheI-1057 bp amplicon-HindIII^3′^ was gel purified and cloned into the *luciferase* reporter, the promoter-less pGL3 basic vector (Promega, Madison, WI) between Nhe I-HindIII sites to generate the wild-type (WT) miR-210 promoter-reporter construct spanning -1030 bp to +31 bp relative to the transcriptional start site (Supplementary Figure 1). Bio-informatics (Genomatix) analyses of this miR-210 promoter region identified three putative HIF-1α/ARNT heterodimer binding sites or HREs with core elements of these HREs at -446 (HRE1), -402 (HRE2), -63 bp (HRE3), and a fourth site with core element at -7 where Myc-associated zinc finger protein related transcription factor binds (Supplementary Figure 1). Site specific deletions of one binding site at a time created four different deletion constructs, namely Δ1, Δ2, Δ3 and D4.

### Reporter gene assays to test deletion constructs of HREs in miR-210 promoter

The WT-miR-210-pGL2 promoter-reporter construct and all the four deletion constructs were prepared endotoxin free and sequences were confirmed. Reporter gene assays were performed using H9c2 cells as previously described [22]. H9c2 cells were transiently co-transfected with promoter-reporter construct plus internal control pRLSV40-Luc vector using the X-treme GENE HP DNA transfection reagent (Roche, Indianapolis, IN) for 20 hours at 37°C. Cells were then exposed to either normoxia (21% O_2_) or hypoxia (1% O_2_) for 26 hours in fresh medium at 37°C. *Firefly* and *Renilla reniformis* luciferase activities in cell lysates were measured in a Modulus Microplate Luminometer (Turner Biosystems, Sunnyvale, CA) using a dual-luciferase reporter assay system (Promega). Promoter activities were then calculated by normalizing the *firefly* luciferase activities (pGL3 constructs) to *Renilla reniformis* luciferase activity of pRL-SV40-Luc vectors. Final data were expressed as luciferase activity relative to WT210-pGL3 construct in normoxia control.

### Electrophoretic mobility shift assays (EMSA)

Nuclear extracts were isolated from fetal rat hearts using the NXTRACT Cell Lytic Nuclear Extraction Kit (Sigma-Aldrich). The oligonucleotide probes representing three putative HRE motifs and Site 4 in the rat miR-210 promoter were labeled with biotin using the Biotin 3′-end labeling kit followed by EMSA assays using LightShift Chemiluminescent EMSA kit (Pierce Biotechnology, Rockford, IL) as previously described [22]. Briefly, single stranded oligos were incubated with Terminal Deoxynucleotidyl Transferase (TdT) and biotin-11-dUTP in binding mixture for 30 minutes at 37°C. The TdT adds a biotin-labeled dUTP to the 3′-end of the oligonucleotides. The oligos were extracted using chloroform and isoamyl alcohol to remove the enzyme and unincorporated biotin-11-dUTP. Dot blots were performed to ensure the oligos were labeled equally. Combination of sense and antisense oligos, heating to 95°C for 2 minutes, followed by overnight cooling at room temperature were sequentially done to anneal complementary oligonucleotides. The labeled oligonucleotides were then subjected to binding reaction with nuclear extracts (NE) in the binding buffer (LightShift kit). Binding reactions were performed at room temperature for 30 minutes in 20 μL containing 50 fmol biotinylated probes, 1X binding buffer, 1 μg of poly (dI-dC), and 10 μg of NE. For cold competition reactions, a 200-fold molar excess of unlabeled homologous oligonucleotides were also added to binding reactions. For supershift experiments, we adopted the supershipft protocol of Wang *et al*. [59] with minor modifications. First 1 mL of anti-HIF-1α antibody (Active Motif, Carlsbad, CA) or 1 mL normal rabbit sera were incubated with NE (10 μg) in binding buffer for 2 hours at 4°C. Next, the binding reaction was performed by adding the biotin-labeled probe as described above. The samples were then electrophoresed on a non-denaturing 5% polyacrylamide gel/0.5X TBE in the cold room. The contents of the polyacrylamide gel were then transferred to a nylon membrane (Pierce) and oligos were crosslinked to the membrane using a UV crosslinker (125 mJoules/cm^2^). Finally, supershifted bands were visualized using photographic films using the protocol and reagents provided in the Light Shift kit (Pierce).

### Plasmid constructs and reporter gene assays to detect miR-210-GR mRNA interaction

A 223 bp segment of 3′-UTR of rat GR mRNA harboring the prospective target region of mature miR-210-3p was first PCR amplified from normal rat heart cDNA using the fo0rward (5′-gagacccCTCGAGggctagacacccattttcaca) and reverse (5′-gagacccTCTAGAgggctactactgcttctgttttg) primers designed based on the rat GR mRNA sequence (accession #: M14053.1) but containing artificial XhoI (CTCGAG) and XbaI (TCTAGA) sites in forward and reverse primers respectively to facilitate cloning. Subsequently, the XhoI-223bpGR 3′UTR-XbaI fragment was cloned between XhoI (5′) and XbaI (3′) sites in the pmiRGLO luciferase vector (Promega) to generate the pmiRGLOXGRX reporter construct used in the reporter gene assay, following a methodology similar to Zhang *et al*. [60]. For experimental validation of the miR-210-3p target in rat GR 3′-UTR, H9c2 cells were transfected with 500 ng/well empty pmiRGLO (pmirGLO control vector), pmiRGLOXGRX construct only, pmiRGLOXGRX plus 7 nM miR-210 mimic (miR-210) (Qiagen) or 7 nM scrambled miRNA mimic (Qiagen) plus Attactene transfection reagent (Qiagen) following methodologies as described by Qiagen. After 48 hours, the *Firefly* and *Renilla reniformis luciferase* activities in cell extracts were measured in a luminometer using a dual-luciferase reporter assay system (Promega). The relative *Firefly* luciferase activity was normalized with *Renilla reniformis luciferase* activity and expressed as relative to control pmiRGLO activity (% control) as described previously [61].

### Western blotting

The whole cell proteins were extracted from cultured cardiomyocytes following treatments as previously described [54, 56, 62]. In brief, whole cell lysates were prepared using RIPA lysis buffer supplemented with 2% (v/v) protease cocktail inhibitors, 2 mM phenylmethanesulfonylfluoride and 1 mM sodium orthovanadate (Santa Cruz Biotechnology, Santa Cruz, CA). Lysates were incubated for 1 hour on ice, followed by centrifugation at 14,000 rpm for 10 minutes at 4°C and supernatant collection for further use. Protein concentrations were measured using the BCA protein assay kit (Pierce). Equal amounts of proteins were separated by SDS-PAGE and transferred to polyvinylidene fluoride (PVDF) membranes. Membranes were blocked with 5% non-fat milk in TBS buffer for 1 hour at room temperature, and then incubated with primary antibodies against GR (Cell Signaling, Danvers, MA) or GAPDH (Abcam, Cambridge, MA) overnight at 4°C. After a brief wash, membranes were then incubated with horseradish peroxidase-conjugated secondary antibodies (Santa Cruz Biotech). Protein signal was visualized with enhanced chemiluminescence reagents (Pierce) and blots were exposed to Hyperfilm. The results were scanned and analyzed with NIH image J software. Protein levels of GR were detected and normalized to GAPDH levels.

### Quantitative RT-PCR for GR or miR-210 levels

Total RNA was isolated from whole fetal hearts or cultured cardiomyocytes using TRIzol reagent (Life Technologies). Isolated RNA was converted to cDNA using the SuperScript III First-Strand Synthesis SuperMix (Life Technologies) for GR mRNA detection, or miScript II RT kit (Qiagen) for miR-210 detection according to the manufacturer's instructions. GR mRNA levels were detected using the iQ SYBR Green Supermix (Bio-Rad, Hercules, CA). Primers included: GR, Forward: AGGTCTGAAGAGCCAAGAGTTA; Reverse: TGGAAGCAGTAGGTAAGGAGAT; and Actin: Forward: TCAGGTCATCACTATCGGCAAT; Reverse: ACTGTGTTGGCATAGAGGTCTT. MiR-210 levels were detected using the miScript SYBR Green PCR kit (Qiagen). Primers included miScript Universal Primer, miR-210 miScript Primer Assay (Rn_miR-210_1; Cat#MS00000644; Qiagen) and SNORD61 miScript Primer Assay (Hs_SNORD61_11; Cat#MS00033705; Qiagen). PCR was done in triplicate and threshold cycle numbers were averaged for each sample. The values were expressed as fold of normoxia.

### Cell culture-based hypoxia-reoxygenation injury and annexin-V staining for apoptosis detection

Primary fetal cardiomyocytes or H9c2 cells were incubated in a hypoxic (1% O_2_, 37°C, 5% CO_2_) chamber for 24 hours in the absence of FBS to mimic hypoxic-ischemic injury. Cells were then supplemented with FBS and returned to a normoxic (21% O_2_, 37°C, 5% CO_2_) incubator for an additional 24 hours to mimic reoxygenation prior to assessment of apoptosis. For studies involving the inhibition of the GR, cells were either treated 48 hours prior to hypoxic insult with 1 μM RU486 (Sigma-Aldrich). For siRNA-mediated knockdown of the GR, H9c2 cells were transfected with 100 nM scramble oligo (All Stars Negative, Qiagen) or siRNAs 36 hours prior to hypoxia-reoxygenation treatment. Thus, transfection proceeded for a total of 72 hours prior to assessment of cell death. Efficiency of GR knockdown was evaluated under normoxic conditions using two separate anti-GR oligos (Dharmacon, Lafayette, CO) by qPCR and Western blot. The sequences for these oligos were as follows: Oligo 1 = 5′-ggaaugagaccagauguaa; Oligo 2 = 5′-uuacaaagauugcagguau. Oligos were combined during hypoxia-reoxygenation injury. Cardiomyocyte apoptosis was determined with Annexin-V staining using the Annexin-V-FITC apoptosis kit for flow cytometry (Thermo Fisher Scientific) according to the manufacturer's instructions. Samples were immediately run on a MACSQuant flow cytometer (Miltenyi Biotec, San Diego, CA) and analyzed using the FlowJo software (TreeStar, Ashland, OR).

### Data and statistical analysis

Data were expressed as mean ± SEM. Experimental number (n) represents fetus from different dams. *In vitro* experiments are done at least in triplicate. Comparisons between two groups were analyzed using Student's *t* test (unpaired, 2-tailed). Comparisons between multiple groups were analyzed using ANOVA followed by Newman Keul's *post hoc* test, where appropriate. *P* value less than 0.05 was considered significant.

### Materials

M199 medium was obtained from Hyclone (Logan, UT); MiRNA-210 mimic, HiPerFect transfection reagent, miScript II RT kit, miScript Primer Assay and miScript SYBR Green PCR kit were purchased from Qiagen (Valencia, CA). MiR-210 locked nucleic acid (miR-210-LNA) were purchased from Exiqon (Woburn, MA). RU486, 2-methoxyestradiol, bromodeoxyuridine and NXTRACT Cell Lytic Nuclear Extraction Kit were purchased from Sigma-Aldrich (St. Louis, MO). TRIzol reagent, SuperScript III First-Strand Synthesis SuperMix and Annexin-V-FITC apoptosis kit were obtained from Life Technologies (Carlsbad, CA); The Dual-Glo Luciferase Assay System and pmiRGLO luciferase vector were purchased from Promega (Fitchburg, WI). GR siRNAs were purchased form Dharmacon (Lafayette, CO). The iQ SYBR Green Supermix were purchased from Bio-Rad (Hercules, CA). X-treme GENE HP DNA transfection reagent were purchased from Roche (Indianapolis, IN). RIPA lysis buffer and HRP-conjugated secondary antibody were purchased from Santa Cruz Biotechnology (Santa Cruz, CA). BCA protein assay kit and enhanced chemiluminescence reagents were purchased from Pierce (Rockford, IL). Anti-GR primary antibody were purchased from Cell Signaling (Danvers, MA) and anti-GAPDH primary antibody from Abcam (Cambridge, MA).

## SUPPLEMENTARY MATERIALS FIGURES



## Abbreviations

2ME: 2-methoxyestradiol
3′-UTR: 3′-untranslated region
BrdU: bromodeoxyuridine
GR: glucocorticoid receptor
HAS: HIF-1 ancillary sequence
HBS: HIF-1 binding site
HIF-1α: hypoxia-inducible factor 1-alpha
HREs: hypoxia response elements
IUGR: intrauterine growth restriction
LNA: locked nucleic acid
miR-210: microRNA-210
