# Supplementary material for: MicroRNA-210 suppresses glucocorticoid receptor expression in response to hypoxia in fetal rat cardiomyocytes
Source: Oncotarget. 2017 May 11;8(46):80249–64. doi: 10.18632/oncotarget.17801 (PMC5655194; doi:10.18632/oncotarget.17801)
Supplement: Supplementary file 1 [file oncotarget-08-80249-s001.pdf]

MicroRNA-210 suppresses glucocorticoid receptor expression in response to hypoxia in fetal rat cardiomyocytes

Supplementary Materials

```
      5′-
      CCAGAAGAGGACATCAGATCCCATTACAGATGGTTGTGAGCCA
      CCATGTGGTTGCTGGGAATTGAACTCAGGACTTTTGGAAGTCA
      GTGCTCTTAACCACTGAGCCATCTCTCCAGCCCCGCAGCTGCT
      GCATCTTATTGAGTGATTGAGAGCTAAATGCCCTATGTTTTCT
      CCACAGGAACAACCTGGGGTGATGGAAGTGTGTAGGGAAGGAG
      GTCTCTTCTGGCTGCACCTCCATCGCACTGCTTTGGCTCTCCT
      CCTAGGAGTCCAATAAATGACCTTGGAACTTTGGTCTATTTCT
      CAAAAGACAGGGGCAAGCCCTTTGAGAGCCCAGCGCTCAACGG
      GACTCTCCCTCCAAGAATAGATCGCTTACACGGAGTGGAGCGA
      CAAAGGCAAATCTCACCGTAGAGAAGGGAGTCCCCGAGGCCAA
      ACCCCTAAGAGCTCCAGGCAAGTCAGAGACAGAACCCCTTCTC
      ACAGCCAGATGGACACATATCTTTCAGGATCTACAGGGTCTGG
HRE1  AGAAAGGCAGAGGCGTACGTGCCTCCCGAGGGCACGGTGTCCC
HRE2  TGGCCTTCCAGGCCACCCCTGGGCCATTGCCAGGAATACGTGC
      GCCTGGAGAGTTGTGAGTTCCCGAAGAGGAGCAGTTCTTCGAC
      TGGGTAGGGGAATAACTGACCCGCCTAAACTTCAGTCTGGGAG
      GCGGGGCTGTTGCTTCTTTGCCTCAAAGGGCCTCCCTTGCCCA
      GCTGTCTCATTGCGCTGCCCCGGTGAAGCATCCCACTATCAGG
      GACCAGAGCCAACTCAACCCAAATGCAGAAAGCCTGGGCCGTA
      CCACCGCTCGAGGGAAGGACATGCCTTGGATTGTACCAACGCC
      CAATCACAGGGAGACACTGTCCAGCGAGGCCGCCCTGCAGGCC
☆HRE3 CCGCCCAGCAGTGGCAGCCCCTCCCCAAGCCGACGTGCAGAAA
      AGAACGCGCCGGGCTCGCCCGCGAATGGCTTGCTCGGCGGCGG
Site4 GCGGGGTGT GATGCCTCCCCCTCGTGTTCACTGGCTGCAG
                +1
```

Supplementary Figure 1: Rat miR-210 promoter sequence. Three putative HRE sites for HIF-1α/ARNT dimer binding were identified by bioinformatics search. Binding sites are boxed and core elements in HRE sites are indicated in red. Site 4 is shown in blue. +1 = transcription initiation site (based on RNA-Seq data, NCBI).

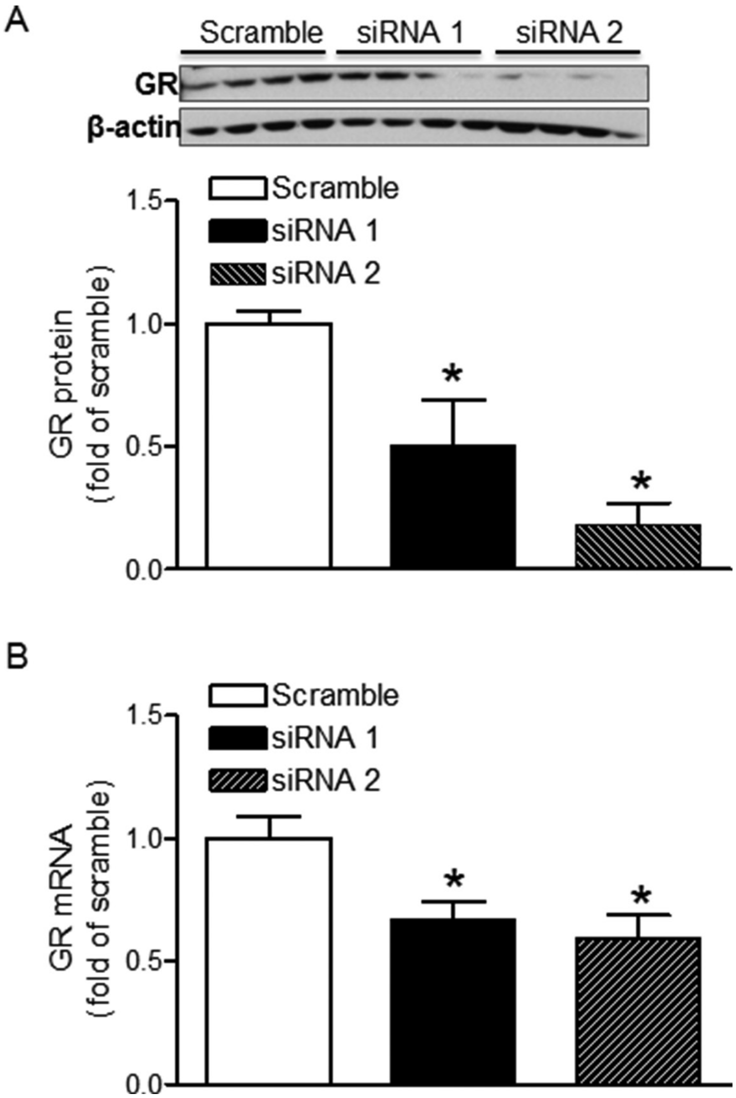

Supplementary Figure 2: GR knockdown by GR siRNAs. H9c2 cells were transfected with two GR siRNA oligos for 72 hours. The levels of GR protein (**A**) and mRNA (**B**) were determined by Western blot or qPCR. Data are mean ± SEM. *$p < 0.05$, siRNA vs. scramble; $n = 4$.
